# A simulation optimization method for coordination of production, transportation and sales

**DOI:** 10.1016/j.fmre.2023.06.013

**Published:** 2023-09-09

**Authors:** Yi Zheng, Ming Lei, Yijie Peng

**Affiliations:** Guanghua School of Management, Peking University, Beijing 100871, China

**Keywords:** Supply chain coordination, Marketing strategy, Simulation optimization, Linear programming, Mixed integer linear programming

## Abstract

This study considers a problem of coordinating production, transportation and sales in a multi-echelon supply chain network. A simulation model is built to generate the random customer demands at different locations, which are affected by a marketing strategy. Customer demands need to be satisfied by the supply chain through production, transportation and distribution. The optimization problem for coordination of production, transportation and distribution is first formulated as a linear programming with demands as input parameters in the constraint. Our objective is to maximize the expectation of the optimal profit of the supply chain given random demands by selecting an optimal marketing strategy. A simulation optimization technique is proposed to control the generation of random demands and solve the linear programming for efficiently learning the optimal marketing strategy. Numerical results show that our method can significantly improve the expected profit of the supply chain and reduce the computational burden of solving linear programming for achieving a given level of probability of correct selection of the optimal marketing strategy. Furthermore, we extend the optimization problem to a mixed integer programming and also demonstrate the computational efficiency of our proposed method.

## Graphical abstract

**Figure:**
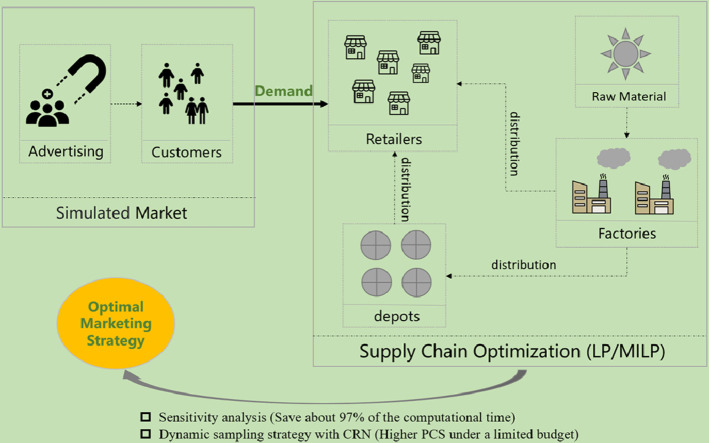

## 1 Introduction

The supply chain is a functional network chain connected by suppliers, manufacturers, distributors and end users. It includes a complete manufacturing process from raw materials to intermediate products and final products, and finally, the products are delivered to consumers by the sales network. Production, transportation and sales are the key components of supply chain, and coordinating them effectively is of great significance for improving the overall management decision-making and helping enterprises cope with supply chain risks such as supply shortage and demand fluctuation [1].

However, traditionally, each stage of the supply chain is optimized independently, which ignores the potentially conflicting objectives of different components. For example, reducing the number of goods shipped to the warehouse can decrease the cost of inventory, but it also reduces the economies of scale in transportation, which leads to higher transportation costs. To improve the performance of supply chain, different problems have been considered for coordinating the components of supply chain, such as the production-inventory problem [[2], [3]] and inventory-transportation problem [[4], [5]]. They can be divided into three categories in terms of modeling [6]: (1) mathematical programming model [[7], [8], [9], [10]]; (2) economic model [11]; (3) simulation model [[12], [13]]. In the first category, the most common modeling approaches are mixed integer linear programming (MILP) and mixed integer nonlinear programming (MINLP), which require many unrealistic assumptions, such as known demands and no backorders [[14], [15]].

Although Leitch [16] finds that taking marketing strategy into account for production planning can smooth seasonal fluctuations in demand and adjust production plans and thus significantly increase profits, few studies have rigorously integrated the marketing strategy into modeling for designing supply chain systems. In most literature of supply chain management, sales is considered as a transportation process of delivering products to customers or retailers, which does not capture the effect of a marketing strategy [[17], [18], [19]].

A well-designed marketing strategy can reduce mismatch between supply and demand by influencing the purchasing behavior of customers. Yet a high-fidelity demand generation model that captures causality between marketing and purchasing behavior has rarely been introduced in supply chain system optimization. Kaplan et al. [20] consider the impact of price elasticity changes on demand, and further analyze its impact on the operation of the supply chain. The authors find that meeting customers demand precisely rather than producing more than necessary would result in an increase in system revenue, but they need to make restriction on the absolute value of price demand elasticity to avoid difficulties in modeling and computing.

Vidal and Goetschalckx [15] point out that mathematical programming techniques cannot accurately represent complicated supply chain networks, so other optimization techniques such as simulation and heuristic approaches may be needed. A recent trend gaining steam is to introduce digital twins in modeling for supply chain management [[21], [22]], namely digital twin-driven supply chain (DTSC) [23]. DTSC has been applied in smart manufacturing to accurately represent the details of a complicated real-world supply chain so that better production decision could be made. Simulation is an important technology for building digital twin systems, which can represent complex dependencies between different agents. Studies show that a combination of simulation modeling, optimization, and data analytics is essential to build a DTSC [[21], [24], [25]].

The existing literature mainly focuses on how to use traditional methods of supply chain management (SCM) in DTSC. For example, mathematical programming is used to determine the optimal coordinates for the location of new facilities (warehouses, distribution centers), and simulation allows details of production sites and logistics infrastructure facilities to be incorporated in modeling [21]. Baruffaldi et al. [26] design a warehouse management simulation system to illustrate how mathematical programming and simulation techniques work together to support warehousing decisions in the system. For supplier selection, Cavalcante et al. [27] first establish a simulation model containing four suppliers, and then use the output data of the simulation model (including supplier delivery time and delivery quantity) to train a machine learning model which produces supplier selection decision, in turn evaluated by the simulation model.

Our study combines simulation and optimization techniques to coordinate production, transportation, and sales. The marketing simulation modeling is based on system dynamics, specifically, a Bass diffusion model with advertising effect, which generates random customer demands to be satisfied by a multi-echelon supply chain network through production, transportation and distribution. The optimization problem for the supply chain is first formulated as a linear programming with demands as input parameters in the constraint. Our objective is to maximize the expectation of the optimal profit of the supply chain given random demands by selecting an optimal marketing strategy. The linear programming (LP) model determining production, transportation and distribution decisions together for supply chain management is often large scale. Moreover, it requires a large number of simulations to accurately estimate the expected optimal profit under a given marketing strategy, so the computational burden would typically be very high. Through the sensitivity analysis for the constraint parameters in LP, we can see that the basis matrix and dual solution would not change when the random demand generated by simulation lies in an interval. Therefore, when estimating the expected optimal profit, it is possible to accurately find the optimal marketing strategy by solving the LP given a small number of simulated demands. We provide an upper bound of error as a function of the sampling size. Simulation samples sequentially generated for competing strategies gradually reveal which strategy is more likely to be the optimal one. Therefore, an intelligent dynamic sampling strategy can effectively improve the efficiency of selecting the optimal marketing strategy. Given a fixed number of simulation samples, the computational burden for achieving a given level of probability for correctly selecting the optimal marketing strategy can be reduced by using common random numbers (CRN) that creates positive correlation between simulation outputs of different strategies. Furthermore, we extend the optimization problem for the supply chain to a MILP by considering a location selection problem, and our objective is also to maximize the expectation of the optimal profit of the supply chain given random demands by selecting an optimal marketing strategy. Analogously, the optimal marketing strategy can be accurately obtained by solving the MILP given a small number of simulated demands through sensitivity analysis, and the dynamic sampling scheme with CRN further improves the efficiency of selecting the optimal marketing strategy.

The rest of the paper is organized as follows: Section 2 formulates the problem. Section 3 proposes an algorithm and conducts theoretical analysis for accelerating techniques. Section 4 extends the problem to a MILP and proposes the corresponding algorithm. In section 5, numerical experiments are conducted to compare computational efficiency and correct selection probability of the proposed algorithm with other algorithms under different market scenarios. We conclude the paper in Section 6.

## 2 Problem statement

**Fig. 1:**
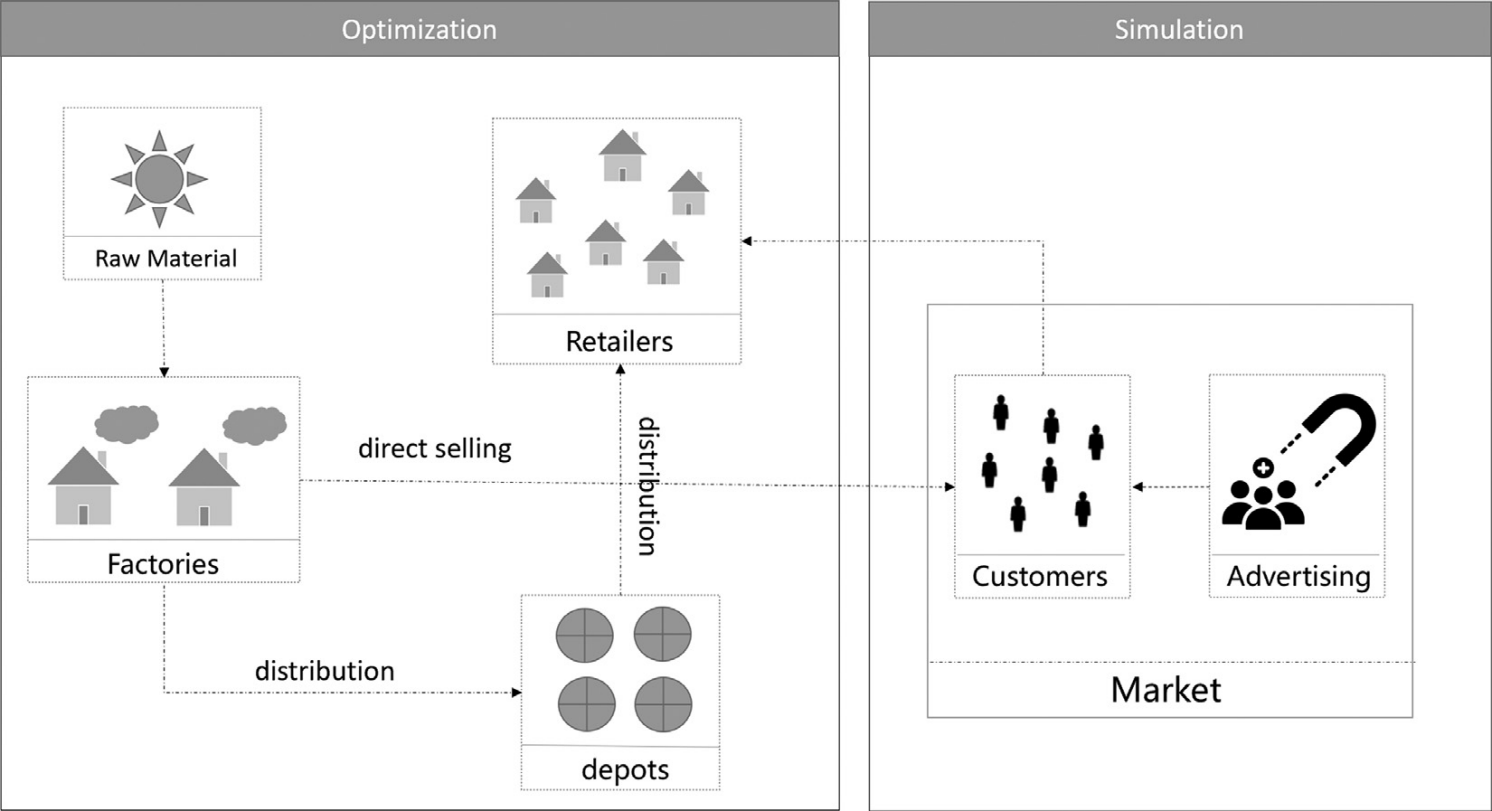
**Structure of production, transport, and sales coordination problem**.

This paper studies a problem of coordinating production, transportation and sales in a multi-echelon supply chain network, where random customer demand is dependent on the market strategy. Given a set of suppliers, warehousing and transshipment centers, and retailers, the profit of the supply chain is maximized given the constraint that the random customer demand is satisfied. Fig. 1 shows the structure of our problem, which consists of two components: (1) a random process of demand generation affected by the marketing strategy, depicted by a systems dynamics simulation model; and (2) optimization of supply chain operations given the demand. The purpose of this paper is to efficiently achieve the integrated optimization of the above two components.

### 2.1 Simulation model for demand generation

In reality, seasonal factors can affect the demand of many products or services, including hotels [28], food [29], and clothing [30]. Demand is also affected by marketing strategies [[31], [32]].

In addition, with the rapid development of social networking, product recommendation behavior in social interaction has attracted the attention of researchers in the field of marketing [33]. Studies have found that consumers’ purchase choices are directly influenced by others’ behaviors [34], especially the recommendations from people in social networks [35], which is also known as word-of-mouth (WOM) marketing.

It may be more economical for companies to retain existing customers than to attract new ones since the latter cost is usually much higher. Studies have shown that old customers often have certain switching barriers when they change product/service providers [36]. Even though unsatisfied with the existing product/service, they may continue to choose it because of the potential psychological cost for switching [37]. The marketing simulation model capturing the effect of WOM and switching barriers in this study is set as follows:

There are a certain number of potential consumers in the market initially, whose demand is influenced by the marketing strategy. The demand quantity of each customer is 1 each time. Once the demands of potential consumers are met, they become actual users, and these actual users influence the purchase decisions of potential consumers through WOM. When the product service life is over, users will become potential consumers with purchasing needs. The amount of demand at each moment is dependent on the marketing strategy, the length of the product service life, and the seasonal demand intensity of the product. Table 1 shows the related parameters and their meanings.

**Table 1:** **Table of simulation parameters and meanings**.

Parameters	Meanings
Ad	Advertising intensity of a market strategy
PU	Initial number of potential consumers
w	Number of potential consumers influenced by a user through WOM
s	Ratio of current seasonal demand to average demand
l	Length of product service life

According to Eskin and Baron [38], there is a significant positive linear relationship between advertising expenditure and demand. The impact of WOM on demand varies among different products, but on average, there is a positively linear correlation with sales amount [39]. For example, Chevalier and Mayzlin [40] study the impact of online reviews and user-generated content (UGC) on book sales and find that there is a significant positive correlation between the logarithm of the number of user reviews and the logarithm of book sales.

The seasonal fluctuation patterns of demand for different products are different. Common seasonal fluctuation patterns include unimodal, bimodal, and hockey effects, and we follow the seasonal demand pattern in ref. [41], where the demand for products peaks either in the fall or the month before Christmas, or both.

The quantity of new demand $Nd_{t}$ for products at time t is determined by the number of potential consumers $PU_{t}$, number of actual users $U_{t}$ at time t, the advertising intensity Ad, the random WOM effect w, and the degree of seasonal fluctuation $s_{t}$ at time t as follows:

$$Nd_{t}=\left(PU_{t}\cdot Ad+\frac{PU_{t}}{PU_{t}+U_{t}}\cdot U_{t}\omega \right)\cdot s_{t}+C_{1}$$

where $C_{1}$ is a constant set to 0 in our numerical experiment. This means that the minimum quantity of new demand at time t is equal to 0. Assume that the time t when the users purchasing the product is uniformly distributed on the timeline, the number of products whose service life ends at t is $Q_{t}$ which equals to the number of actual users $U_{t}$ at time t divided by the length of product service life l:

$$Q_{t}=\frac{U_{t}}{l}$$

The total product demand $d_{t}$ at time t is affected by two aspects: (1) the new demand $Nd_{t}$ for the product at time t, and (2) the old consumers’ renewed demand $Q_{t}$ at time t. Thus we have

$$d_{t}=\left(Nd_{t}+Q_{t}\right)\cdot t+C_{2}$$

where $C_{2}$ is a constant representing the initial demand in the market. It can be set to any value not less than 0. The number of actual users $U_{t}$ at time t is given by

$$U_{t}=-Q_{t}\cdot t+C_{3}$$

where $C_{3}$ is a constant representing the initial number of users in the market, which can be set to any number not less than 0.

**Fig. 2:**
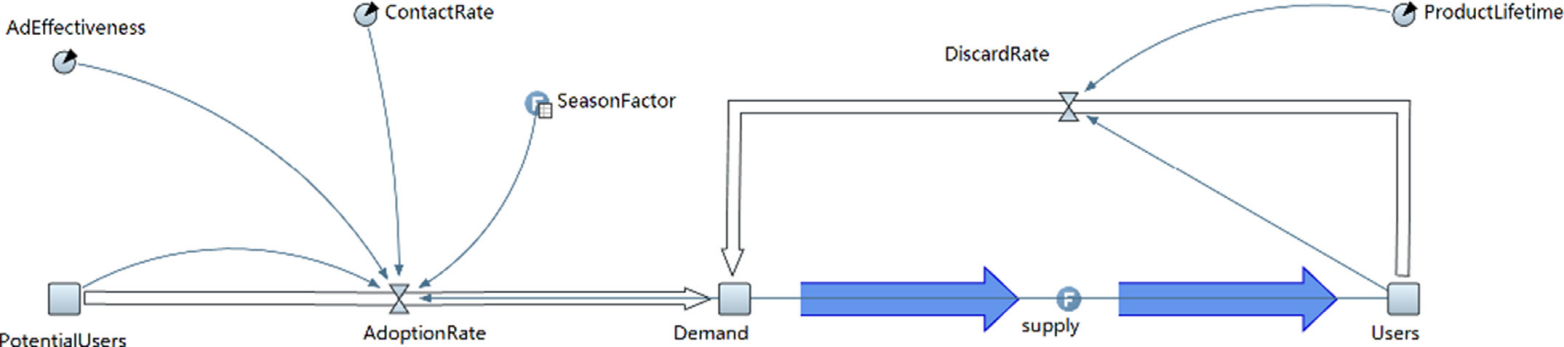
**Demand system dynamics**.

The demand changes dynamically over time according to the mechanism above, and the system dynamics process is shown in Fig. 2.

Given a marketing strategy determined by parameter Ad, consumer demand is generated randomly, and then these demands are satisfied by the supply chain through production, transportation and distribution. Our objective is to maximize the expected optimal profit of the supply chain with respect to marketing strategy parameter Ad.

### 2.2 Supply chain optimization under given demand

A supply chain network usually includes suppliers, distribution centers and retailers. The supply chain optimization problem considered in this paper is described as follows. We define a complete graph G=(N,A), where the node set N contains the supplier set F={1,F}, the warehousing and transshipment center set D={1,D}, and the retailer set R={1,R}, and the arc set A={(i,j):i,j∈N,i≠j} represents the logistics transportation route. After the product is manufactured in the supplier’s factory, it is first shipped to the warehousing and transshipment center, and then shipped to the retailer or directly to the retailer. Consumers at different locations buy products from their nearest retailer. Each factory cannot produce more than its maximum capacity, and each warehousing and transshipment center can only store and transfer products up to certain limits. Given parameters including unit transportation cost, product sales price, and factory maximum capacity, the optimization problem is to determine the production quantity of the supplier’s factory, the distribution quantity to the warehousing and transshipment center, the retailer, and the route of product transportation, so as to maximize the sales profit. The parameters and decision variables are listed in Table 2.

**Table 2:** **Optimization model parameters and variables**.

Parameters	
$c_{st}$	unit cost of transportation from s to t
p	unit product price
$ms_{f}$	maximum supply of supplier f
$mt_{d}$	maximum transshipment quantity of warehousing transshipment center d
${\tilde{b}}_{r}$	random demand for the product at retailer r
$c_{Ad_{z}}$	cost of adopting marketing strategy z
c	unit production cost
h	unit holding cost
Decision Variables	
$x_{st}$	product quantity shipped from s to t
$s_{f}$	production quantity of supplier f

Therefore, the optimization problem of coordinating production, transportation and marketing under uncertain demand can be formulated as the following LP problem:

(1) $$\begin{array}{c} \max\sum_{r\in R}p{\tilde{b}}_{r}-\sum_{(s,t)\in N\times N}c_{st}x_{st}-\left(\sum_{f\in F}s_{f}-\sum_{r\in R}{\tilde{b}}_{r}\right)h-\sum_{f\in F}cs_{f} \end{array}$$

s.t.

(1.1) $$\sum_{f\in F}s_{f}\geq \sum_{r\in R}{\tilde{b}}_{r}$$

(1.2) $$\sum_{t\in N}x_{ft}=s_{f},\;\forall f\in F$$

(1.3) $$s_{f}\leq ms_{f},\;\forall f\in F$$

(1.4) $$\sum_{s\in N}x_{sr}={\tilde{b}}_{r},\;\forall r\in R$$

(1.5) $$\sum_{s\in N}x_{sd}=\sum_{t\in N}x_{dt},\;\forall d\in D$$

(1.6) $$\begin{array}{c} \sum_{s\in N}x_{sd}\leq mt_{d}.\;\forall d\in D \end{array}$$

The objective function 1 is to maximize sales profit. Eq. 1.1 ensures that the total product produced by the supplier’s factory can meet the demands of customers at all retailers. The inventory balance and the maximum production capacity of the supplier are guaranteed by Eqs. 1.2 and 1.3, respectively. Eq. 1.4 is the supply and demand balance constraint between product distribution and customer demand. Eq. 1.5 ensures the total product flow coming in must equal the total flow going out and Eq. 1.6 ensures the maximum throughput capacity at each warehousing and transshipment center is not violated.

The optimal marketing strategy is to maximize the expectation of the profit in supply chain with respect to uncertain demands for a given marketing strategy as follows:

(2) $$\begin{array}{ccc} \max_{Ad_{z}\in AD} & & E_{{\tilde{b}}_{r}}[\max\sum_{r\in R}p{\tilde{b}}_{r}-\sum_{(s,t)\in N\times N}c_{st}x_{st}\\ & & -\;\left(\sum_{f\in F}s_{f}-\sum_{r\in R}{\tilde{b}}_{r}\right)h-\sum_{f\in F}cs_{f}]-c_{Ad_{z}} \end{array}$$

where AD is the marketing strategy set and $Ad_{z}$ represents marketing strategy z.

## 3 Integrated optimization of simulation and mathematical programming

### 3.1 Efficiency enhancement for solving optimization

#### 3.1.1 Sensitivity analysis for linear programming

We conduct sensitivity analysis for LP to facilitate solving optimization problem 2. Suppose some component $b_{r}$ of the demand vector b is changed to $b_{r}+\delta$, or equivalently, the vector b is changed to $b+\delta e_{r}$, where $e_{r}$ is the rth unit vector. There is a range of δ, within which the current basis remains optimal [42], i.e.,

(3) $$B^{-1}\left(b+\delta e_{r}\right)\geq 0$$

where B is an optimal basis for the original problem. Let $g=(\beta_{1r},\beta_{2r},\beta_{mr})$ be the rth column of $B^{-1}$, Eq. 3 becomes

(4) $$x_{B(j)}+\delta \beta_{jr}\geq 0,\;j=1,...,m$$

where m is the number of columns of B, $\beta_{jr}$ is the element of the jth row and the rth column of $B^{-1}$, and $x_{B(j)}$ is the element of the jth row of the column vector $B^{-1}b$. Eq. 4 is equivalent to

(5) $$\max_{\left\{j|\beta_{jr}>0\right\}}\left(-\frac{x_{B(j)}}{\beta_{jr}}\right)\leq \delta \leq \min_{\left\{j|\beta_{jr}<0\right\}}\left(-\frac{x_{B(j)}}{\beta_{jr}}\right)$$

For δ in this range, the optimal objective function value obj can be expressed as a linear function of δ, i.e., $obj=p^{\prime }b+\delta p_{r}$, where $p^{\prime }=c_{B}^{\prime }B^{-1}$ is the (optimal) dual solution associated with the current basis B, $p_{r}$ is the dual solution component corresponding to the constraint governed by $b_{r}$ in the original LP problem 1. If δ is within the allowed range determined by Eq. 5, the demand $b_{r}+\delta$ corresponds to the same basis matrix B, and the value coefficient vector $c_{B}$ corresponding to the basic variables is also unchanged, so the dual solution $p^{\prime }$ is unchanged. Therefore, there is no need to solve the LP problem for all randomly generated demands.

#### 3.1.2 Simulation optimization

Evaluating the performance of a marketing strategy via real-world experiment would be typically expensive. Using computer modeling and simulation can greatly reduce the evaluation cost of marketing strategies. Ranking and selection (R&S) method can further improve the computational efficiency of selecting the optimal strategy from different marketing strategies through simulation. In our simulation model introduced in Section 2, the demand given a market strategy is random. The R&S algorithm selects the best marketing strategy $Ad_{o}$ from the k alternative market strategies $\left\{Ad_{z},z=1,...,k\right\}$ whose mean performance $\left\{\mu_{z},z=1,...,k\right\}$ have no analytical form and can only be estimated by simulation. Since simulation budget is often limited, how to allocate simulation replications intelligently among these k marketing strategies to improve the efficiency of correctly selecting the optimal strategy is the key issue to address. Introducing positive correlations among competing designs would typically increases probability of correct selection (PCS). To achieve this, we let the WOM parameters across strategies be generated with the same random seeds. The CBA (Correlated Budget Allocation) algorithm is an efficient sampling strategy that maximizes the PCS based on correlated simulation outputs given a simulation budget constraint [43]. We introduce some notations used in the algorithm.

T= total number of simulation replications (budget),

k= number of designs (strategies in our research),

$N_{i}=$ number of simulation replications allocated to design i,

$\mu_{i}=$ mean of design i,

$\sigma_{i}^{2}=$ variance of design i,

$C_{ij}=$ covariance between paired replications of design i and j ($=\sigma_{i}^{2}$ for j=i),

$\rho_{ij}=C_{ij}/\left(\sigma_{i}\sigma_{j}\right)$, correlation between paired replications of design i and j,

$\alpha_{i}={\left(\mu_{1}-\mu_{i}\right)}^{2}$, where we assume $\mu_{1}$ is the mean of the best design without loss of generality,

Ω={2,...,k}, set containing all indices except the best.

The algorithm is sketched as follows. Given the total number of simulation replications T, the number of simulation replications for the best design $N_{1}^{*}$ is determined by

(6) $$T/\left[1+\sum_{i\in J\cup I\setminus I(M)}\frac{\sigma_{i}^{2}}{\alpha_{i}M-\sigma_{1}^{2}+2C_{1i}}+\sum_{i\in I(M)}\frac{\sigma_{1}^{2}-2C_{1i}}{\alpha_{i}M-\sigma_{1}^{2}}\right]$$

where $I=\{i\in \Omega |2\rho_{1i}<\sigma_{i}/\sigma_{1}\}$ and $J=\Omega \setminus I=\{i\in \Omega |2\rho_{1i}\geq \sigma_{i}/\sigma_{1}\}$,

$$M=\arg\min_{x\geq A_{0}}\frac{h(x)}{T}$$

$$A_{0}\equiv \left(\max_{i\in J}A_{i}\right)\vee \left(\max_{i\in I}\frac{\sigma_{1}^{2}}{\alpha_{i}}\right)$$

where $A_{i}=\frac{\sigma_{1}^{2}+\sigma_{i}^{2}-2C_{1i}}{\alpha_{i}},i=2,...,k$, a∨b≡max(a,b).

$$h\left(x\right)\equiv x+\sum_{i\in J\cup I\setminus I(x)}\frac{\sigma_{i}^{2}x}{\alpha_{i}x-\sigma_{1}^{2}+2C_{1i}}+\sum_{i\in I(x)}\frac{\left(\sigma_{i}^{2}-2C_{1i}\right)x}{\alpha_{i}x-\sigma_{1}^{2}}$$

$$I\left(x\right)=\left\{i\in I|A_{i}>x\right\}$$

After $N_{1}^{*}$ is determined by Eq. 6, the optimal allocations of other strategies i∈Ω are determined by

(7) $$N_{i}^{*}=\left\{ \begin{array}{c} \frac{\sigma_{i}^{2}}{\alpha_{i}M-\sigma_{1}^{2}+2C_{1i}}N_{1}^{*}\leq N_{1}^{*}\\ if\;2\rho_{1i}\geq \sigma_{i}/\sigma_{1}\;or\;A_{i}<M\\ \frac{\sigma_{1}^{2}-2C_{1i}}{\alpha_{i}M-\sigma_{1}^{2}}N_{1}^{*}\geq N_{1}^{*}\;otherwise \end{array}\right.$$

Theorem 3.1 *The demands generated under different market strategies using CRN for the WOM parameter are positively correlated, and the objectives in*1*under different market strategies are also positively correlated.*

The proof of Theorem 3.1 can be found in Appendix A. Next, we design algorithms separately for two scenarios. Scenario 1 is the targeted marketing scenario. The marketing activities are carried out for a single retailer in certain location, so when the marketing strategy changes, only the demand of the targeted retailer is affected. Scenario 2 is a network marketing [44] scenario. When the marketing strategy changes, the demands at all retailers are affected.

### 3.2 Algorithms for two marketing scenarios

#### 3.2.1 Targeted marketing scenario

We simulate the model in Section 2 n times given a marketing strategy in $\left\{Ad_{z},z=1,...,k\right\}$, which generates random demands $\left\{b_{1}^{z},b_{n}^{z}\right\}$, where the maximum value is $b_{\max}$ and the minimum value is $b_{\min}$. We need to calculate the optimal solutions of LP 1 for all generated demands $\left\{b_{1}^{z},...,b_{n}^{z}\right\}$.

Based on the property of the LP problem, we first calculate the optimal solution of LP given demand $b_{i}^{z}$ and the corresponding range given by 5. If there is another demand $b_{j}^{z}$ whose variation $\delta_{j}^{z}=b_{j}^{z}-b_{i}^{z}$ relative to $b_{i}^{z}$ lies in the range given by Formula 5, then the optimal solution would not change and perturbation of the objective function is $\delta_{j}^{z}p_{i}$, where $p_{i}$ is the component of the dual solution corresponding to the constraint governed by $b_{i}^{z}$. If none of the other simulated demand is in the range given by Formula 5 corresponding to $b_{i}^{z}$, we solve the LP problem given another demand, which could be done efficiently by dual simplex method based on the outputs of the simplex method for solving the LP problem given demand $b_{i}^{z}$. We can efficiently solve all LP problems with n random demands, because many of them are solved as byproducts of solving other LP problems with only minimal computational overhead. Then the objective value in Formula 2 of a given market strategy is estimated by the sample average of the objective values in 1 for all simulated random demands.

For efficiently solving optimization problem 2 via simulation, we apply the R&S method in Section 3.1.2 to allocate a total of simulation budget N for estimating the objective function 2 of all market strategies. The unknown parameters in Formulas 6-7 can be estimated by $n_{0}$ initial simulation replications equally allocated to each marketing strategy. Let $N_{z}$ be the number of simulation replications calculated by Formulas 6-7 for marketing strategy $Ad_{z}$. Then the additional ${\left(N_{z}-n_{0}\right)}^{+}$ simulation replications are allocated to getting a more accurate estimate of the objective in Formula 2 for a marketing strategy $Ad_{z}$. We present this scheme in Algorithm 1.

**Algorithm 1:**
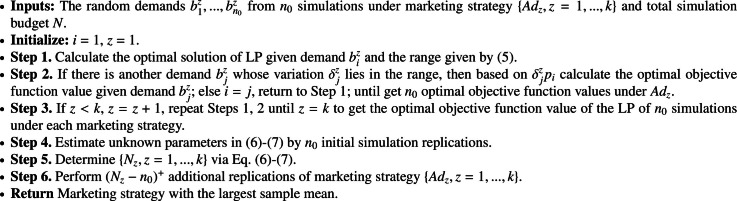
**Targeted Marketing**.

#### 3.2.2 Network marketing scenario

In the network marketing scenario, the constraint vector $b^{i}$ obtained from the ith simulation corresponds to multiple (not necessarily all) components in the constraint vector b of the LP problem in Section 2.2. Before proposing the algorithm for the network marketing scenario, we first prove that the following lemma holds:

Lemma 3.1 *When there are multiple components in the constraint vector*b*that change at the same time, as long as the change ratio*$\alpha_{v}=\frac{\Delta b_{r}}{b_{r}}$*of the variation*$\Delta b_{r}$*to the original value of the changing component*$b_{r}$*is the same and belongs to the following interval:*

(8) $$\underset{r\in V}{⋂}\left[\max_{\left\{j|\beta_{jr}>0\right\}}\left(-\frac{x_{B(j)}}{x_{B_{V}\left(j\right)}}\right),\min_{\left\{j|\beta_{jr}<0\right\}}\left(-\frac{x_{B(j)}}{x_{B_{V}\left(j\right)}}\right)\right]$$

*then the current optimal basis matrix*B*does not change, where*V*is the set of the subscripts of changing components,*$\beta_{jr}$*is the element of the*j*th row and the*r*th column of*$B^{-1}$*, and*$x_{B_{V}}=B^{-1}b_{V}$*, where*$b_{V}$*is a column vector whose elements are*$b_{r}$*or 0 when the component in*b*is unchanged.*

Proof The current optimal basis matrix B remains unchanged when $B^{-1}\left(b+\alpha_{v}b_{V}\right)\geq 0$ holds, which can be rewritten as

$$x_{B(j)}+\alpha_{v}x_{B_{V}\left(j\right)}\geq 0,\;j=1,...,m$$

where $x_{B(j)}$ is the element of the jth row of the column vector $B^{-1}b$, $x_{B_{V}\left(j\right)}$ is the element of the jth row of the column vector $x_{B_{V}}$. When the change ratio $\alpha_{v}$ of the component $b_{r}$ belongs to the following interval:

$$\max_{\left\{j|\beta_{jr}>0\right\}}\left(-\frac{x_{B(j)}}{x_{B_{V}\left(j\right)}}\right)\leq \alpha_{v}\leq \min_{\left\{j|\beta_{jr}<0\right\}}\left(-\frac{x_{B(j)}}{x_{B_{V}\left(j\right)}}\right)$$

the basis matrix B remains optimal. Considering all changing components, we have the allowed range of change ratio $\alpha_{v}$:

$$\underset{r\in V}{⋂}\left[\max_{\left\{j|\beta_{jr}>0\right\}}\left(-\frac{x_{B(j)}}{x_{B_{V}\left(j\right)}}\right),\min_{\left\{j|\beta_{jr}<0\right\}}\left(-\frac{x_{B(j)}}{x_{B_{V}\left(j\right)}}\right)\right]$$

 □

Similarly, we can also derive the case when all the components in b are changed.

Corollary 3.1 *When all components in*b*change at the same time, as long as the change ratio*$\alpha =\frac{\Delta b}{b}$*of each component*b*is the same and greater than or equal to -1, the current optimal basis*B*does not change.*

Proof The current optimal basis matrix B remains unchanged when $B^{-1}\left(b+\alpha b\right)\geq 0$ holds, or

$$x_{B(j)}+\alpha x_{B(j)}\geq 0,j=1,...,m$$

which leads to the conclusion:

α≥−1

 □

We generate n random demand vectors $b_{1}^{z},b_{n}^{z}$ based on a model in Section 2 given a marketing strategy in $\left\{Ad_{z},z=1,...,k\right\}$, where the maximum and minimum values of $b_{ir}^{z}$ for i∈{1,...,n} are $b_{r}^{\max},b_{r}^{\min}$, respectively. We need to calculate the optimal solutions of LP 1 for all generated demands $b_{1}^{z},b_{n}^{z}$.

Based on Lemma 3.1, we first calculate the optimal solution of LP given demand vector $b_{i}^{z}$ and the corresponding range given by Formula 8. If there is another demand vector $b_{j}^{z}$ whose component $b_{jr}^{z}$’s change ratio $\alpha_{j}^{z}=\frac{b_{jr}^{z}-b_{ir}^{z}}{b_{ir}^{z}}$ relative to $b_{ir}^{z}$ lies in the range given by 8, then the optimal solution would not change and perturbation of the objective function is $\delta_{j}^{z\prime }p_{i}$, where $p_{i}$ is the dual solution vector corresponding to the constraint governed by $b_{i}^{z}$ and $\delta_{j}^{z}$ is a vector with component $\delta_{jr}^{z}=\left(b_{jr}^{z}-b_{ir}^{z}\right)$. Similar procedure as in Section 3.2.1 can be implemented to estimate the objective value in Formula 2 efficiently for a given market strategy.

We then apply the R&S method in Section 3.1.2 to efficiently solve optimization problem Formula 2 by sequentially allocating a total of simulation budget N to estimating the objective function 2 for all market strategies. The unknown parameters in Formulas 6-7 are estimated by $n_{0}$ initial simulation replications equally allocated to each marketing strategy. Let $N_{z}$ be the number of simulation replications calculated by Formulas 6-7 for marketing strategy $Ad_{z}$. Then the additional ${\left(N_{z}-n_{0}\right)}^{+}$ simulation replications are allocated to getting a more accurate estimate of the objective in Formula 2 for a marketing strategy $Ad_{z}$. We present this scheme in Algorithm 2.

**Algorithm 2:**
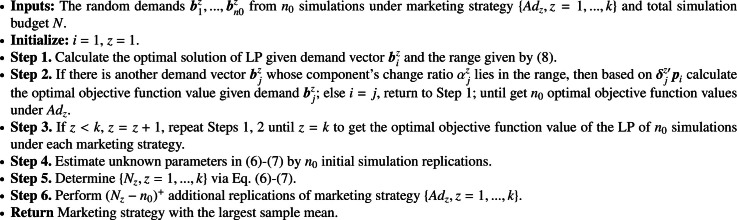
**Network Marketing**.

### 3.3 Analysis of algorithms

In this section, we analyze the error of our algorithm under finite simulation budget. Here we introduce some assumptions before the analysis.

Assumption 3.1 There are upper and lower bounds $\left[{\underset{‾}{b}}_{r},{\bar{b}}_{r}\right]$ of customer demand $b_{r}$ at each retailer.

Assumption 3.2 $b_{r}$ follows a unimodal distribution, i.e., there is only one optimum in its distribution. The variable corresponding to the optimum is M, and the probability density function is denoted as $g(b_{r};M)$, abbreviated as $g(b_{r})$.

Assumption 3.3 The problem Formula 2 in Section 2.2 has the optimal solution, denoted as PB, PB≥0 and $PB≜\max_{Ad_{z}\in AD}E_{{\tilde{b}}_{r}}\left[\max\sum_{r\in R}p{\tilde{b}}_{r}-\sum_{(s,t)\in N\times N}c_{st}x_{st}\right]-c_{Ad_{z}}.$

Assumption 3.4 Changes in network marketing strategies lead to the same change ratio $\alpha_{v}$ of customer demand $b_{r}$ at different retailers, which is only related to the marketing strategy $Ad_{z},z=1,...,k$.

Assuming that in $n_{0}$ simulations of a single retailer’s demand under a targeted marketing strategy $Ad_{z}$, we obtain $n_{s}$ different ranges $\left\{\delta_{n}=\left[b_{n}+{\underset{‾}{\delta }}_{n},b_{n}+{\bar{\delta }}_{n}\right],n=1,...,n_{s}\right\}$ where the optimal basis matrix B remains unchanged in the range, and ${\bar{\delta }}_{n}$, ${\underset{‾}{\delta }}_{n}$ are the upper and lower bounds of the range given by Formula 5 corresponding to $b_{n}$, respectively. Define $\Delta b_{\min}=\min_{n=1,...,n_{s}}\left|\delta_{n}\right|$, where $|\delta_{n}|={\bar{\delta }}_{n}-{\underset{‾}{\delta }}_{n}$.

Theorem 3.2 *The error rate*$\epsilon_{z}$*by Algorithm 1 satisfies the following equation:*

(9) $$\epsilon_{z}\leq g\left(M\right)\cdot \left({\bar{b}}_{r}-{\underset{‾}{b}}_{r}-n_{s}\Delta b_{\min}\right)$$

Proof According to Assumption 3.3, the problem 2 has an optimal solution PB, so the sampling error $\epsilon_{Ad_{z}}$ under the marketing strategy $Ad_{z}$ is $PB\cdot (1-\int_{\cup_{n=1}^{n_{s}}\delta_{n}}g\left(b_{r}\right)db_{r})$, and the error rate

(10) $$\begin{array}{ccc} \epsilon_{z} & = & \frac{PB\cdot (1-\int_{\cup_{n=1}^{n_{s}}\delta_{n}}g\left(b_{r}\right)db_{r})}{PB}\\ & = & 1-\int_{\cup_{n=1}^{n_{s}}\delta_{n}}g\left(b_{r}\right)db_{r}\\ & = & \int_{{\underset{‾}{b}}_{r}}^{{\bar{b}}_{r}}g\left(b_{r}\right)db_{r}-\int_{\cup_{n=1}^{n_{s}}\delta_{n}}g\left(b_{r}\right)db_{r}\\ & = & \int_{\left[{\underset{‾}{b}}_{r},{\bar{b}}_{r}\right]\setminus \cup_{n=1}^{n_{s}}\delta_{n}}g\left(b_{r}\right)db_{r} \end{array}$$

Since the sum of the lengths of $n_{s}$ minimum ranges must be less than or equal to the sum of the lengths of all ranges, i.e., $n_{s}\Delta b_{\min}\leq \left|\cup_{n=1}^{n_{s}}\delta_{n}\right|$, hence

$${\bar{b}}_{r}-{\underset{‾}{b}}_{r}-n_{s}\Delta b_{\min}\geq \left|\left[{\underset{‾}{b}}_{r},{\bar{b}}_{r}\right]\setminus \cup_{n=1}^{n_{s}}\delta_{n}\right|$$

Since $g\left(M\right)\geq g\left(b_{i}\right)$, then we have $g\left(M\right)\cdot \left({\bar{b}}_{r}-{\underset{‾}{b}}_{r}-n_{s}\Delta b_{\min}\right)\geq \int_{\left[{\underset{‾}{b}}_{r},{\bar{b}}_{r}\right]\setminus \cup_{n=1}^{n_{s}}\delta_{n}}g\left(b_{r}\right)db_{r}=\epsilon_{z}$. □

**Fig. 3:**
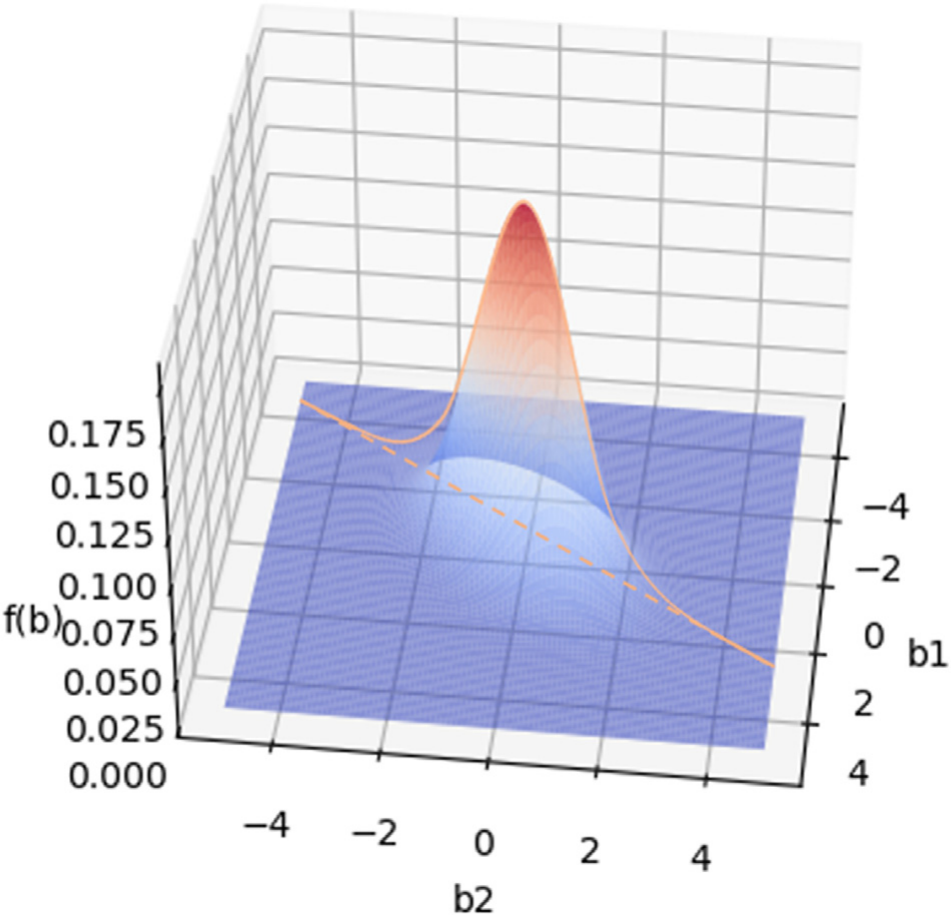
**Two-dimensional unimodal distribution probability density**.

According to Assumption 3.4, for a fixed demand $b_{r}$ at retailer r, the demand at other retailers is determined accordingly under network marketing strategies. Taking two retailers as an example, as shown in Fig. 3, we see that the joint distribution of random variables $b_{1}$ and $b_{2}$ constitutes a one-dimensional unimodal distribution which is denoted as $g(b_{r}^{\prime })$, and its maximum is denoted as $M_{r}^{\prime }$.

In $n_{0}$ simulations of multiple retailers’ demand under a networking marketing strategy $Ad_{z}$, $n_{s}^{\prime }$ different ranges $\left\{\delta_{n}^{\prime }=\left[b_{n}^{\prime }+{\underset{‾}{\alpha }}_{v_{n}}b_{n}^{\prime },b_{n}^{\prime }+{\bar{\alpha }}_{v_{n}}b_{n}^{\prime }\right],n=1,...,n_{s}^{\prime }\right\}$, where the basis matrix B remains optimal in the range, are obtained, and ${\bar{\alpha }}_{v_{n}}$, ${\underset{‾}{\alpha }}_{v_{n}}$ are the upper and lower bounds of the range given by 8 corresponding to $b_{n}^{\prime }$, respectively. Define $\Delta b_{\min}^{\prime }=\min_{n=1,...,n_{s}^{\prime }}\left|\delta_{n}^{\prime }\right|$, where $|\delta_{n}^{\prime }|=b_{n}^{\prime }\cdot \left({\bar{\alpha }}_{v_{n}}-{\underset{‾}{\alpha }}_{v_{n}}\right)$. Then we have the following:

Theorem 3.3 *The error rate*$\epsilon_{z}^{\prime }$*by Algorithm 2 satisfies the following equations:*

(11) $$\epsilon_{z}^{\prime }\leq g\left(M_{r}^{\prime }\right)\cdot \left({\bar{b}}_{r}-{\underset{‾}{b}}_{r}-n_{s}^{\prime }\Delta b_{\min}^{\prime }\right)$$

*The proof is similar to*Theorem 3.2*, so the detail is omitted.*

### 3.4 Adaptability to different contexts

This paper essentially presents an algorithmic framework combining mathematical programming and simulation optimization, supporting all scenarios that can be similarly modeled as mathematical programming problems and simulation optimization problems. Specifically, the general steps to use this framework are as follows:

**Step1.** Analyze the influencing factors of random variables, and form different simulation alternatives (marketing strategies in this paper) under the conditions of different influencing factors.

**Step2.** Construct an LP/MILP model of the problem, whose parameters are random variables influencing by factors in simulation model.

**Step3.** Select the algorithm (such as Algorithm 1) according to the type of problem modeling (LP or MILP) and the number of components change (1 or more than 1) in simulation model.

#### 3.4.1 An illustrative example

We consider a flight pricing and human resource allocation problem. The number of flight attendants required for each time period on a specific route is shown in Table 3, and is determined by the number of passengers and the type of aircraft at that time. Generally speaking, the more passengers there are, the more flight attendants are needed. The aircraft type is also affected by the final number of passengers, and the number of passengers is strongly correlated to the ticket price [45]. Assume that the flight attendants go to work at the beginning of each time period and work continuously for 8 h. The objective is to optimize the air ticket price and reasonably arrange the flight attendants on this route to meet the work needs and maximize the benefits.

**Table 3:** **Flight attendants requirements by time periods**.

Time	Number of Flight Attendants Required
06:00−10:00	$y_{1}$
10:00−14:00	$y_{2}$
14:00−18:00	$y_{3}$
18:00−22:00	$y_{4}$
22:00−02:00	$y_{5}$
02:00−06:00	$y_{6}$

In the above human resource allocation problem, the number of flight attendants required for a certain period of time is the random variables. Depending on the range of factors considered, this problem can be modeled as an LP or MILP problem.

First, the key influencing factor on the number of flight attendants is the air ticket price, which is also easily controllable by airlines. Therefore, different pricing strategies can be used as simulation alternatives. Similar to the steps in Section 2.1, consider the factors that may affect the number of flight attendants and use them to establish a simulation model. The simulation model should generate an output of the number of flight attendants and be capable of running under different pricing strategies.

Second, construct this problem as an LP problem E1, and use the simulation results above as input parameters for the constraints of the established LP model.

(E1) $$\max\sum_{i=1}^{6}f_{i}n_{i}-\sum_{i=1}^{6}w_{i}x_{i}$$

s.t.

(E1.1) $$x_{1}+x_{6}\geq {\tilde{y}}_{1}$$

(E1.2) $$x_{1}+x_{2}\geq {\tilde{y}}_{2}$$

(E1.3) $$x_{2}+x_{3}\geq {\tilde{y}}_{3}$$

(E1.4) $$x_{3}+x_{4}\geq {\tilde{y}}_{4}$$

(E1.5) $$x_{4}+x_{5}\geq {\tilde{y}}_{5}$$

(E1.6) $$x_{5}+x_{6}\geq {\tilde{y}}_{6}$$

where $x_{i}$ is the decision variable, representing the number of flight attendants starting work in the ith time period. $f_{i}$, $n_{i}$ and $w_{i}$ are parameters, representing the ticket sales price of the route, the number of passengers, and the wage of the flight attendants in the ith time period, respectively.

Finally, select an algorithm based on the number of constraints affected by the pricing strategy, and perform simulations and related calculations according to the algorithm requirements.

## 4 Extension of supply chain optimization problem

### 4.1 Mixed integer linear programming problem

We further consider location problems in supply chain optimization, and the optimization problem of coordinating site location, production, transportation and marketing under uncertain demand can be constructed as the following MILP problem:

(12) $$\begin{array}{ccc} \max & & \sum_{r\in R}p{\tilde{b}}_{r}-\sum_{(s,t)\in N\times N}c_{st}x_{st}-\sum_{d\in D}e_{d}c_{d}\\ & & -\;\left(\sum_{f\in F}s_{f}-\sum_{r\in R}{\tilde{b}}_{r}\right)h-\sum_{f\in F}cs_{f} \end{array}$$

s.t.

(12.1) $$\sum_{f\in F}s_{f}\geq \sum_{r\in R}{\tilde{b}}_{r}$$

(12.2) $$\sum_{t\in N}x_{ft}=s_{f},\;\forall f\in F$$

(12.3) $$s_{f}\leq ms_{f},\;\forall f\in F$$

(12.4) $$\sum_{s\in N}x_{sr}={\tilde{b}}_{r},\;\forall r\in R$$

(12.5) $$\sum_{s\in N}x_{sd}=\sum_{t\in N}x_{dt},\;\forall d\in D$$

(12.6) $$\sum_{s\in N}x_{sd}\leq mt_{d}\cdot e_{d},\;\forall d\in D$$

(12.7) $$x_{st},s_{f}\in R^{n},\;e_{d}\in \left\{0,1\right\}$$

where $e_{d}$ is a binary decision variable, such that $e_{d}=1$ if the warehousing and transshipment center d is established, and $e_{d}=0$ otherwise.

The optimal marketing strategy is to maximize the expectation of the profit in supply chain with respect to uncertain demands for a given marketing strategy as follows:

(13) $$\begin{array}{ccc} \max_{Ad_{z}\in AD} & & E_{{\tilde{b}}_{r}}[\max\sum_{r\in R}p{\tilde{b}}_{r}-\sum_{(s,t)\in N\times N}c_{st}x_{st}-\sum_{d\in D}e_{d}c_{d}\\ & & -\;\left(\sum_{f\in F}s_{f}-\sum_{r\in R}{\tilde{b}}_{r}\right)h-\sum_{f\in F}cs_{f}]-c_{Ad_{z}} \end{array}$$

### 4.2 Sensitivity analysis for mixed integer linear programming

The sensitivity analysis of an MILP problem with respect to scalar variations of its right-hand side can be described by the following formulation [46]:

(14) $$z\left(\theta \right)=\min_{x,y}c^{T}x+d^{T}y\;\;\;$$

s.t.

(14.1) $$\mathrm{Ax}+\mathrm{Dy}\leq b_{0}+\theta_{V}$$

(14.2) $$x^{l}\leq x\leq x^{u}$$

(14.3) $$\theta_{\min}\leq \theta_{s}\leq \theta_{\max},\;s=1,...,m$$

(14.4) $$x\in R^{n},\;y\in {\left\{0,1\right\}}^{t}$$

where x is a vector of continuous variables, y is a vector of 0-1 binary variables, $\theta_{V}$ is a vector of parameters, $\theta_{s}$ is the sth component of $\theta_{V}$, $\theta_{\min}$ and $\theta_{\max}$ are the lower and upper bounds on $\theta_{s}$. It is assumed that the problem has been already solved at a point $b=b_{0}+\theta_{0}$ with the objective function value $z_{0}$, and the optimal solution obtained was ($x^{*},y^{*}$). If the integer variables are fixed and an LP sensitivity analysis is carried out, we can obtain the θ-limits (corresponding to the range given by Formulas 5 and 8 in targeted and networking scenarios, respectively) and define its upper bound as $\theta^{u}$. The variation of the objective function value as a linear function of the parameter can be expressed as

(15) $$\bar{z}\left(\theta \right)=z_{0}+p^{\prime }\theta$$

where θ is a column vector whose element $\theta_{i}=\theta_{s}-\theta_{0}$ and $\theta_{0}\leq \theta_{s}\leq \theta^{u}$, $\theta_{0}$ is the component of $\theta_{0}$. p is the dual solution corresponding to the constraint governed by θ. The dimensions of p and θ is the number of changing components in b. Eq. 15 provides a parametric upper bound for the minimization problem.

If the integer solution remains optimal in the interval [$\theta_{0},\theta^{u}$], then the LP sensitivity analysis result Formula 15 holds true also for the MILP problem. Thus, the MILP sensitivity analysis problem is posed as that of either identifying a successor integer solution in the interval [$\theta_{0},\theta^{u}$], or accept the LP sensitivity results, i.e., the integer solution $y^{*}$ is optimal in the interval [$\theta_{0},\theta^{u}$]. Therefore, we need to determine whether there is a threshold $\theta^{\prime }$ such that the integer solution remains optimal within $\left[\theta_{0},\theta^{\prime }\right]$.

To identify the successor integer solution in the interval [$\theta_{0},\theta^{u}$], Formula 14 is solved again after the following constraints are imposed

(16.1) $$\theta_{0}\leq \theta_{s}\leq \theta^{u}$$

(16.2) $$c^{T}x+d^{T}y\leq z_{0}+p^{\prime }\theta$$

(16.3) $$\sum_{i\in F^{1}}y_{i}-\sum_{i\in F^{0}}y_{i}\leq \left|F^{1}\right|-1$$

Eq. 16.1 is straightforward, and Eq. 16.2 excludes integer solutions with higher values than the current upper bound. Eq. 16.2 will hold as an equality if there is a vector $\theta^{*}$, at which a new integer solution $y^{\prime }$ just becomes optimal, which means that the value function of the new integer solution is exactly $z_{0}+p^{\prime }\theta^{*}$. The current optimal solution is explicitly precluded by Formula 16.3, where $F^{0}$ and $F^{1}$ are the index sets of the integer variables valued at zero and one respectively, and $|F^{1}|$ denotes the cardinality of $F^{1}$.

The constraints of our maximization problem are as follows

(17.1) $$\theta^{l}\leq \theta_{s}\leq \theta_{0}$$

(17.2) $$c^{T}x+d^{T}y\geq \left(z_{0}+p^{\prime }\theta \right)$$

(17.3) $$\sum_{i\in F^{1}}y_{i}-\sum_{i\in F^{0}}y_{i}\leq \left|F^{1}\right|-1$$

We define Formula 17 by adding constraints Formulas 17.1–17.3 to 12. Note that $\theta_{s}$ is treated as a variable in 17 and its lower bound $\theta^{l}$ is the lower bound identified in the LP sensitivity analysis by fixing the current integer solution $y^{*}$. Eq. 15 provides a parametric lower bound in maximization problems. If 17 is infeasible, then this infeasibility verifies that the LP sensitivity analysis result Formula 15 holds for the MILP problem as well, i.e., the integer solution $y^{*}$ is optimal in the interval [$\theta^{l},\theta_{0}$]. If 17 is feasible with an integer solution $y^{new}$ and $\theta^{\prime }$, then this provides the limit [$\theta^{\prime },\theta_{0}$] of the optimality of the current solution and the successor optimal integer solution $y^{new}$. We can fix the new integer solution $y^{new}$ as before to obtain a new lower bound from the LP sensitivity analysis. After finite iterations of the mechanism above, we obtain Formula 15 in all intervals corresponding to different optimal integer solutions and dual solutions, so there is no need to solve the MILP for all randomly generated demands.

### 4.3 Algorithm for extension problem

The sensitivity analysis of MILP in Section 4.2 provides a general framework for both targeted marketing and network marketing scenarios. In Section 3.2, we have generated random demands $\left\{b_{1}^{z},b_{n}^{z}\right\}$ and $\{b_{1}^{z},b_{n}^{z}$} for the targeted marketing and network marketing scenarios, respectively. We need to calculate the optimal solutions of MILP Formula 12 for all generated demands.

We first calculate the optimal solution ($x_{i}^{*},y_{i}^{*}$) and optimal objective value $z_{i}$ of MILP given demand $b_{i}^{z}$ ($b_{i}^{z}$). We fix the integer solution $y_{i}^{*}$ to perform LP sensitivity analysis and then we can get the corresponding dual solution p and θ-limits given by Formula 5 or 8. Considering the lower bound $\theta_{i}^{l}$ of θ-limits, we add corresponding constraints Formulas 17.1–17.3 to MILP Formula 12 and define it as a new MILP problem Formula 17. If Formula 17 is feasible with an integer solution $y^{new}$ and $\theta_{i}^{\prime }$, we get the new interval with $\theta_{i}^{\prime }$ as the lower bound and the current integer solution is optimal in the interval; if 17 is infeasible, then θ-limits do not change. If there is another demand $b_{j}^{z}$ ($b_{j}^{z}$) whose variation $\theta_{j}^{z}=b_{j}^{z}-b_{i}^{z}$ relative to $b_{i}^{z}$ is within the limits, then the optimal solution would not change and perturbation of the objective function is $p^{\prime }\theta$, where p is the component (vector) of the dual solution corresponding to the constraint governed by $b_{i}^{z}$ ($b_{i}^{z}$). If none of the other simulated demand is within the limits, we solve the MILP problem given another demand. We can efficiently solve all MILP problems with n random demands, because many of them are solved as byproducts of solving other MILP problems with only minimal computational overhead. Then the objective value in Formula 13 of a given market strategy is estimated by the sample average of the objective values in Formula 12 for all simulated random demands.

For efficiently solving optimization problem 13 via simulation, we apply the R&S method in Section 3.1.2 to allocate a total of simulation budget N for estimating the objective function 13 of all market strategies. The unknown parameters in 6-7 can be estimated by $n_{0}$ initial simulation replications equally allocated to each marketing strategy. Let $N_{z}$ be the number of simulation replications calculated by 6-7 for marketing strategy $Ad_{z}$. Then the additional ${\left(N_{z}-n_{0}\right)}^{+}$ simulation replications are allocated to getting a more accurate estimate of the objective in 13 for a marketing strategy $Ad_{z}$. We present this scheme in Algorithm 3.

**Algorithm 3:**
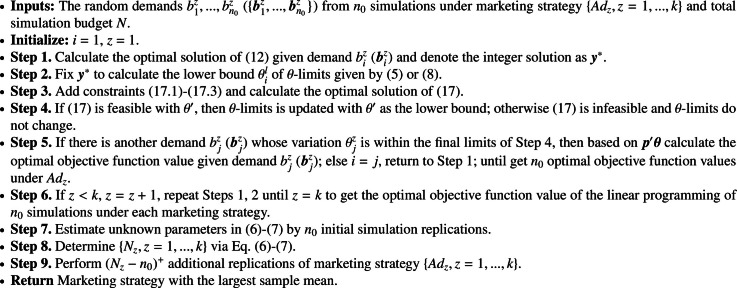
Simulation Optimization Method.

## 5 Numerical results

In this section, we provide numerical results of the MILP model to demonstrate the effectiveness of the algorithms developed in the previous section. The results of the LP model can be found in Appendix C.

### 5.1 Marketing scenario analysis

**Fig. 4:**
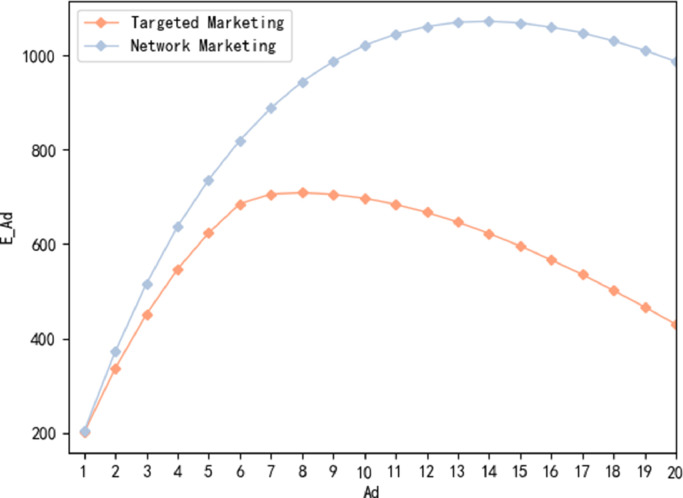
**Expected optimal objective function values under different marketing strategies**.

We consider targeted marketing and network marketing scenarios. Both scenarios include 20 different marketing strategies $\left\{Ad_{i},i=1,...,20\right\}$, the intensity of advertising Ad corresponding to the ith marketing strategy is 0.0001i, and there is a linear relationship between advertising intensity and marketing costs. Initially, there are 100,000 potential consumers in each marketing system, and the number of potential consumers that an actual user can influence through WOM follows a normal distribution $N(0.2+0.1i,0.1^{2}),i=1,...,20$. The specific parameters in the simulation model are shown in Table 4. The demands generated under different marketing strategies are satisfied by a multi-echelon supply chain network consisting of 2 suppliers, 4 warehousing and transshipment centers and 10 retailers, and their parameters are presented in Appendix B. We perform 10,000 simulation experiments under each marketing strategy and take the expected optimal objective function value as the performance of the real system to estimate the PCS in the next section. The expected optimal objective function values of all marketing strategies under the two scenarios are shown in Fig. 4.

**Table 4:** **Parameters setting of the**i**th strategy in simulation model**.

Parameters	Setting
Ad	0.0001i
PU	100,000
w	Normal$\left(0.2+0.1i,0.1^{2}\right)$
s	[0.1,2]
l	Weibull(60,3)

We can see different marketing scenarios lead to significant differences in the corresponding expected optimal objective function values. In general, the profit of network marketing is higher than that of targeted marketing. This is because in network marketing scenarios, although many retailers are affected by marketing, the demand variation of a single retailer is relatively low, and the factories and warehousing and transshipment centers near the retailer can adjust the production plan and the transshipment plan respectively within their capacities to satisfy consumer demands and achieve profit growth. In addition, the optimal marketing investment for targeted marketing strategies is lower than the investment required for the optimal network marketing strategy.

In both marketing scenarios, the optimal marketing strategies are not those with the highest or lowest advertising intensity, but those with moderate marketing investment that attract a certain number of customers. This may be because, when high advertising intensity leads to a surge in demand, not all newly generated demand can be met in a timely manner to achieve sales profits due to constraints in the supply chain, such as production capacity and transportation. On the other hand, while high advertising intensity can attract more customers, it also leads to a sharp increase in marketing costs. This is particularly evident in the scenario of targeted marketing, where there are a large number of retailers (set to 10 in this experiment). It is likely to be uneconomical to invest heavily in targeted marketing for a single retailer alone.

### 5.2 Comparison of computational efficiency

We compare the PCS for three simulation allocation policies: ǣCBAǥ denotes the allocation policy described in Section 3.1.2; ǣIBAǥ denotes the CBA algorithm with zero correlation ($C_{ij}=0$ for all i,j); ǣEBAǥ denotes equal budget allocation, i.e., $N_{1}=N_{2}=...=N_{k}=T/k$. In all cases, k = 20, T = 1000, and $n_{0}$ = 30 for each strategy (600 initial samples total), leaving 400 samples to be allocated in the second stage. Strategies 7 and 11 are the best designs under targeted marketing and network marketing scenarios, respectively, which achieve the largest expected optimal profit.

The correlation is introduced purposely in order to reduce the number of simulation replications for achieving the same PCS level. Specifically, we let the WOM parameters across strategies be generated with the same seeds. Based on some rough estimation analysis, this leads to a correlation around 10%. In the targeted marketing scenario, the mean optimal objective function value varies from 125.5 to 438.7 over the 20 strategies, and the variances vary from 36.3 to 313.5. In the network marketing scenario, the mean optimal objective function value varies from 176.8 to 757.8 over the 20 strategies and the variances vary from 106.1 to 571.3.

**Fig. 5:**
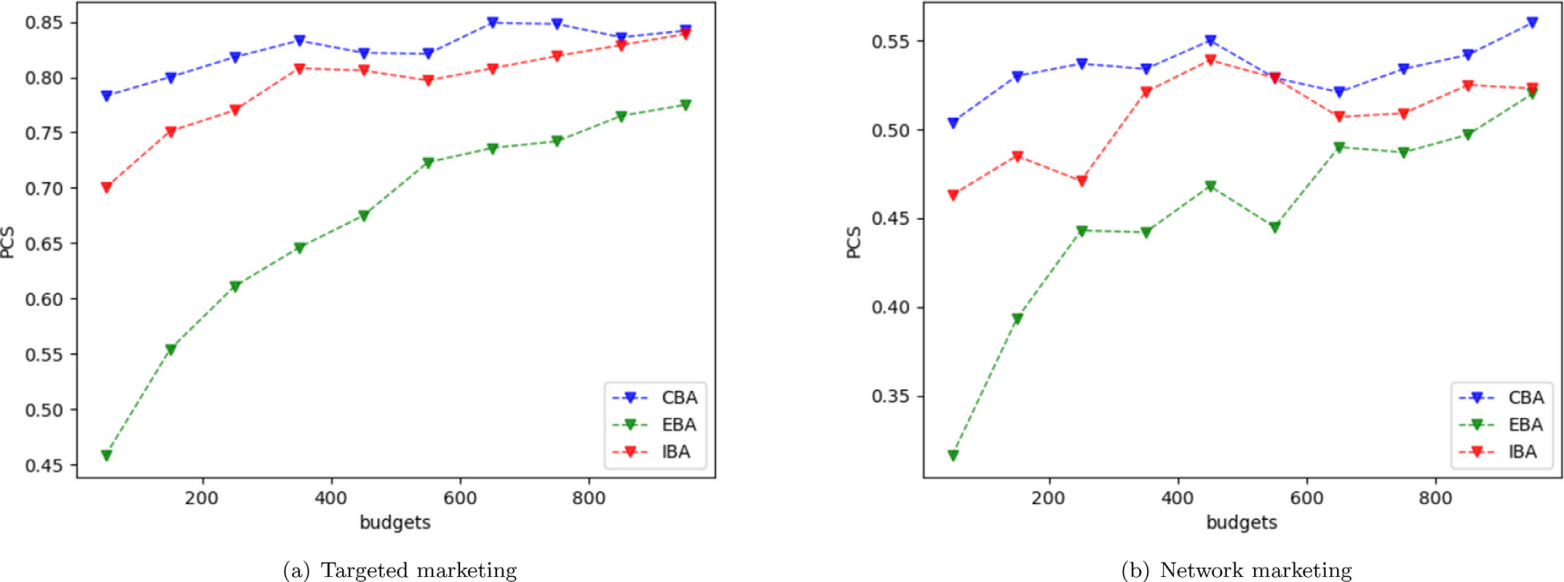
**Estimated PCS under different budgets**.

We first simulate market systems $n_{0}$ times for all strategies under the targeted marketing scenario and network marketing scenario, respectively. Then we use the three algorithms mentioned above to allocate the remaining simulation budget. The experiments are conducted independently, and the estimates of PCS for each algorithm are presented in Fig. 5. The PCS of CBA is higher than EBA and IBA under the constraint of same simulation budget.

We get a total of 2,000 random demands under the two scenarios with T=1000. Accordingly, the optimal solution for 2,000 MILP problems needs to be computed. By performing Algorithm 3, we find that in the targeted marketing scenario, when the demand is supported on [12,134],[134,145],[145,285],[285,434],[434,1892], the corresponding dual variables in Formula 15 in each range do not change, so we need to update the dual variables in Formula 15 and solve Formula 17 5 times separately, and when the demand is supported on [12,285],[286,1892], the corresponding integer solution remains optimal in each range. Since we have solved the MILP problem Formula 12 when Formula 17 has a feasible solution, it only requires to solve the MILP Formula 12 once rather than 1000 times. In the network marketing scenario, when the demand at each retailer is supported on [11,28],[28,63],[63,190], the corresponding integer solution remains optimal in each range, and when the demand is supported on [11,28],[28,38],[38,39],[39,41],[41,63],[63,101], [101,109],[109,119],[119,131],[131,136],[136,151],[151,159],

[159,163],[163,190], the corresponding dual variables in Formula 15 in each range do not change. It only requires to solve the MILP Formula 12 once rather than 1000 times, and update the dual variables in Formula 15 and solve Formula 17 14 times separately. Table 5 compares the computational time of our method with that of the brute-force method, which shows that our method saves 98.56% and 96.39% time in targeted marketing and network marketing scenarios, respectively.

**Table 5:** **Comparison of computation time**.

	Brute-force method	Our Algorithm	Time Saving
Targeted Marketing	32.45s	0.47s	98.56%
Network Marketing	37.92s	1.37s	96.39%

## 6 Conclusion

In this paper, we integrate the marketing strategy into supply chain optimization modeling and propose a simulation optimization technique to coordinate the components of the supply chain. We innovatively combine simulation and optimization methods and demonstrate the superiority of the proposed method in both LP and MILP. In numerical experiments, we compare the computational time of our method with that of the brute-force method, and verify that our method can save about 97% of the computation time in both LP and MILP. We find that in two marketing scenarios, the increase in demand by advertisement might not be fully satisfied due to the constraint of the supply chain, and hence the optimal marketing strategy should well balance the supply chain profit and marketing cost.

## Declaration of competing interest

The authors declare that they have no conflicts of interest in this work.

## Figures and Tables

**Table B.1:** **Parameters table**.

(a) Factory parameters						
Name	Maximum supply					
$f_{1}$	1500					
$f_{2}$	2000					
(b) Warehousing transshipment center parameters						
Name	Maximum transshipment quantity					
$d_{1}$	300					
$d_{2}$	200					
$d_{3}$	300					
$d_{4}$	150					
(c) Product parameters						
Name	Price					
$p_{1}$	3					
c	0.1					
h	0.1					
(d) Transportation cost						
Node	$f_{1}$	$f_{2}$	$d_{1}$	$d_{2}$	$d_{3}$	$d_{4}$
Warehousing transshipment center						
$d_{1}$	0.5	–	–	–	–	–
$d_{2}$	0.5	0.3	–	–	–	–
$d_{3}$	1.0	0.5	–	–	–	–
$d_{4}$	0.2	0.2	–	–	–	–
Retailer						
$r_{1}$	1.5	2.0	1.0	1.0	–	–
$r_{2}$	–	–	1.5	0.5	1.5	–
$r_{3}$	2.5	–	0.5	0.5	2.0	0.2
$r_{4}$	2.0	–	–	1.0	–	1.5
$r_{5}$	–	–	1.5	0.5	0.5	0.5
$r_{6}$	1.5	–	1.0	–	1.5	1.5
$r_{7}$	–	–	1.5	–	0.5	–
$r_{8}$	1.0	–	1.0	–	–	0.5
$r_{9}$	–	–	–	1.0	1.5	–
$r_{10}$	1.5	2.0	–	0.5	–	1.5

1 A ’-’ in the table indicates that the route is not possible, e.g., we cannot ship from $f_{2}$ to $d_{1}$.

**Fig. C.1:**
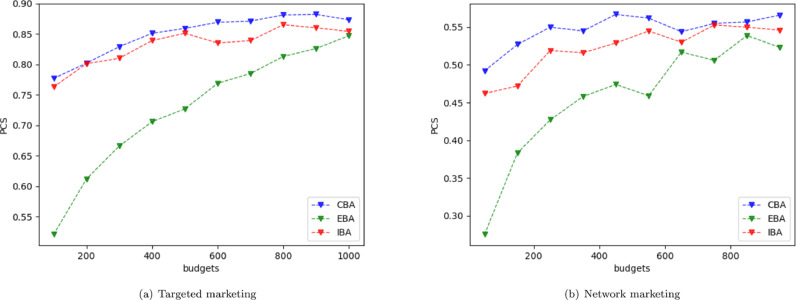
**Estimated PCS under different budgets**.

**Fig. C.2:**
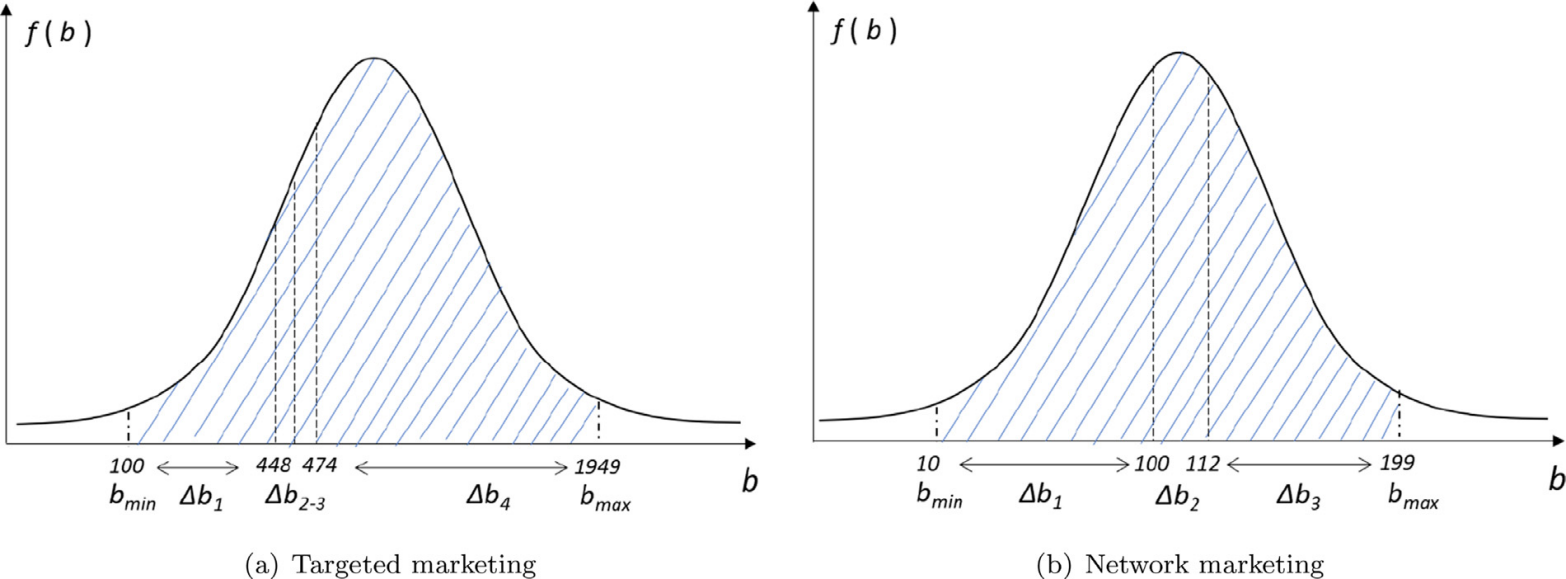
**Random demand probability density plots under different scenarios**.

**Table C.1:** **Comparison of computation time**.

	Brute-force method	Our Algorithm	Time Saving
Targeted Marketing	48.28s	1.52s	96.85%
Network Marketing	47.39s	1.67s	96.48%

## Appendix A Proof of Theorem 3.1

Proof From Section 2.1, the demand can be expressed as a function of the WOM parameter ω as follows:

$$f\left(\omega \right)=\left[\left(\left(PU_{t}\cdot Ad+\frac{PU_{t}}{PU_{t}+U_{t}}\cdot U_{t}\omega \right)\cdot s_{t}+C_{1}\right)+\frac{U_{t}}{l}\right]\cdot t+C_{2}$$

which can be rewritten as

$$f\left(\omega \right)=K_{1}+K_{2}\omega$$

where $K_{1}=\left(PU_{t}\cdot Ad\cdot s_{t}+C_{1}+\frac{U_{t}}{l}\right)t+C_{2}$ and $K_{2}=\frac{PU_{t}}{PU_{t}+U_{t}}U_{t}\cdot t\cdot s_{t}$. According to the parameter definition in Section 2.1, $K_{2}>0$, the demand f(ω) is a positive linear function of ω, so the demands generated using the CRN for the WOM parameter are positively correlated. The objective in 1 can be expressed as a function of the simulated demand $b_{r}$ of the rth retailer as follows:

$$\begin{array}{ccc} h(b_{r}) & = & \max\sum_{r\in R}pb_{r}-\sum_{(s,t)\in N\times N}c_{st}x_{st}-\;\left(\sum_{f\in F}s_{f}-\sum_{r\in R}b_{r}\right)h-\sum_{f\in F}cs_{f}\\ & = & \sum_{r\in R}pb_{r}-\sum_{(s,t)\in N\times N}c_{st}x_{st}^{*}-\;\left(\sum_{f\in F}s_{f}^{*}-\sum_{r\in R}b_{r}\right)h-\sum_{f\in F}cs_{f}^{*} \end{array}$$

where $x_{st}^{*}$ is the optimal solution that maximizes the value of the objective function. According to the definition in Section 2.2, the optimal production quantity should be exactly equal to customer demand, i.e., $\sum_{f\in F}s_{f}^{*}=\sum_{r\in R}b_{r}$ and there is a positive correlation between the quantity shipped and the demand, i.e., $\sum_{(s,t)\in N\times N}x_{st}^{*}=K_{3}\sum_{r\in R}b_{r}$. Given $c_{st}$, $\sum_{(s,t)\in N\times N}c_{st}x_{st}^{*}=K_{4}\sum_{r\in R}b_{r}$, so the equation above can be rewritten as

$$h\left(b_{r}\right)=\left(p-c-K_{4}\right)\sum_{r\in R}b_{r}$$

According to Assumption 3.3, $p-c-K_{4}>0$. There are two simulated demands $b_{r}^{1}$ and $b_{r}^{2}$ $\left(b_{r}^{1}>b_{r}^{2}\right)$, which are scalars in the targeted marketing scenario and vector components in the network marketing scenario. In the network marketing scenario, $b_{r}^{1}>b_{r}^{2}$ means that the component $b_{r}^{1}$ of a simulated demand is greater than the component $b_{r}^{2}$ of another simulated demand, thus we have

$$\frac{h\left(b_{r}^{1}\right)-h\left(b_{r}^{2}\right)}{b_{r}^{1}-b_{r}^{2}}=\frac{\left(p-c-K_{4}\right)(\sum_{r\in R}b_{r}^{1}-\sum_{r\in R}b_{r}^{2})}{b_{r}^{1}-b_{r}^{2}}>0$$

so the objective in 1 is monotone with respect to the demand, and thus the objectives in 1 under different market strategies are also positively correlated. □

## Appendix C Results of the LP model

The simulation experiment setting is consistent with that in Section 5.2, and we also use CBA, IBA, EBA algorithms to allocate simulation budget. The experiments are conducted independently, and the estimates of PCS for each algorithm are presented in Fig. C.1. The PCS of CBA is higher than EBA and IBA under the constraint of same simulation budget.

We get a total of 2,000 random demands under the two scenarios with T=1000. Accordingly, the optimal solution for 2,000 LP problems needs to be computed. Fig. C.2 shows the estimated probability density distribution of random demand obtained by sampling where the boundaries of the shaded area are the upper and lower bounds of $b_{i}^{z}$ obtained by sampling, and the verticals in the shaded area are the upper and lower bounds of the range given by Formula 5 or 8. It can be seen that in the targeted marketing scenario, when the demand is supported on [100,448],[448,461],[461,474],[474,1949], the corresponding optimal basis matrix and dual variables in each range do not change, so it only requires to update the basis matrix for solving the LP 4 times rather than 1000 times. In the network marketing scenario, when the demand at each retailer is supported on [10,100],[100,112],[112,199], we only need to update the basis matrix for solving the LP 3 times rather than 1000 times. Table C.1 compares the computational time of our method with that of the brute-force method, which shows that our method saves 96.85% and 96.48% of time in targeted marketing and network marketing scenarios, respectively.
